# Unimpaired discrimination of fearful prosody after amygdala lesion^☆^

**DOI:** 10.1016/j.neuropsychologia.2013.07.005

**Published:** 2013-09

**Authors:** Dominik R. Bach, René Hurlemann, Raymond J. Dolan

**Affiliations:** a Wellcome Trust Centre for Neuroimaging, University College London, UK; b Zurich University Hospital of Psychiatry, Switzerland; c Department of Psychiatry, University of Bonn, Germany

**Keywords:** Prosody, Fear, Amygdala, Social cognition, Emotion, Urbach–Wiethe

## Abstract

Prosody (i.e. speech melody) is an important cue to infer an interlocutor's emotional state, complementing information from face expression and body posture. Inferring fear from face expression is reported as impaired after amygdala lesions. It remains unclear whether this deficit is specific to face expression, or is a more global fear recognition deficit. Here, we report data from two twins with bilateral amygdala lesions due to Urbach-Wiethe syndrome and show they are unimpaired in a multinomial emotional prosody classification task. In a two-alternative forced choice task, they demonstrate increased ability to discriminate fearful and neutral prosody, the opposite of what would be expected under an hypothesis of a global role for the amygdala in fear recognition. Hence, we provide evidence that the amygdala is not required for recognition of fearful prosody.

## Highlights

• Prosody recognition is assessed in two twin sisters with amygdala lesions due to Urbach–Wiethe syndrome.
• In a multinomial classification task, there is no impairment.
• In a two-alternative forced choice task, patients discriminate fearful and neutral prosody better than a control sample.
• This study provides evidence that the amygdala has no general role in fear recognition.

## 1 Introduction

The perception of a conspecific's emotional state is an important aspect of social communication. In humans this ability relies heavily on non-verbal signals such as facial expression (Ekman & Oster, 1979), emotional speech melody (i.e., prosody) (Banse & Scherer, 1996), and bodily posture (Dael, Mortillaro, & Scherer, 2012).

Extraction of emotional state from a conspecific's facial expression is widely reported to involve the amygdala (Adolphs et al. 1999). Numerous neuroimaging studies have demonstrated amygdala responses to emotional and in particular to fearful expression (Breiter et al. 1996; Fischer et al. 2003; Morris et al. 1996; Whalen et al. 2004; Whalen et al. 1998). Successful identification of fearful facial expression is reported to be impaired following amygdala lesions (Adolphs et al., 1999). This observation could reflect a specific deficit for extraction of emotional meaning from faces, in line with an hypothesised function of the amygdala in face processing, encompassing, but extending beyond, emotional meaning (Atkinson & Adolphs, 2011). On the other hand it is possible that a function of the amygdala includes extraction of information about a conspecific's emotional state, independent of its source.

Here, we capitalised on another source of emotional information, emotional prosody (i. e. speech melody), and investigated whether its identification was impaired in two patients with amygdala lesions. As yet, the role of the amygdala for extraction of emotional meaning from prosody is unclear. Some functional magnetic resonance imaging studies have reported amygdala responses to emotional prosody (Bach et al., 2008; Dolan, Morris, & de Gelder, 2001; Ethofer et al. 2009; Fruhholz, Ceravolo, & Grandjean, 2012; Fruhholz & Grandjean, 2013; Grandjean et al. 2005; Mothes-Lasch, Mentzel, Miltner, & Straube, 2011; Wiethoff, Wildgruber, Grodd, & Ethofer, 2009), but not to fearful voices in particular. Most lesion studies report cases with either unselective, or incomplete, amygdala damage. Impaired fear prosody recognition has been observed in patients with unselective bilateral (Adolphs & Tranel, 1999; Brierley, Medford, Shaw, & David, 2004; Sprengelmeyer et al. 1999) and unilateral (Brierley et al., 2004; Dellacherie, Hasboun, Baulac, Belin, & Samson, 2011) temporal lobe damage, and in one patient with selective, but incomplete, bilateral amygdala resection (Scott et al., 1997). On the other hand, unimpaired fear prosody recognition has been reported in cases with unselective unilateral temporal lobe lesions (Adolphs & Tranel, 1999; Adolphs, Tranel, & Damasio, 2001) or unilateral selective amygdala combined with contralateral extended temporal lobe lesion (Anderson & Phelps, 1998). Furthermore, a large 3D lesion mapping study has shown no clear contribution of medial temporal cortex to prosody recognition (Adolphs, Damasio, & Tranel, 2002), although this might be biased by sampling of lesions. In summary, both the impairments and the heterogeneity of results could reflect lesions to temporal lobe structures outside the amygdala which were differentially affected in the different samples, due to their underlying aetiology (surgical lesions, hippocampal sclerosis, paraneoplastic encephalitis, stroke, and others). Hence, a case of bilateral selective amygdala lesion (SM) showing no impairment in emotional prosody identification might be taken as the most specific finding to date (reported together with other cases in (Adolphs & Tranel, 1999)).

However the small sample sizes studied necessarily entails low power, Further, all studies to date have relied on accuracy measures, i.e. hit rates in a multinomial classification task. This is a common approach in emotion recognition studies which has long been criticised due to a lack of control for false alarms (Wagner, 1993). In an extreme example, a person indiscriminately labelling all stimuli as “angry” will appear impaired in all other emotions, but not in the “angry” category. Or a person with reduced sensitivity to distinguish fearful expression, but with increased bias to label any expression as fearful, might not show any impairment because the preponderance of false alarms, evenly distributed across all other emotion categories, might not exceed the noise level in the control population.

Hence, we sought to extend previous findings reported on patient SM (Adolphs & Tranel, 1999) in three ways: first by examining two further patients with focal amygdala lesions due to congenital Urbach–Wiethe disease; second by using a more powerful and precise metric for prosody identification, namely by means of a two-alternative forced choice task which allows for independent analysis of sensitivity and bias (or criterion) as prescribed by signal detection theory. Finally, because impairments might not be detected due to floor or ceiling effects when normal performance is very low (as for fear in Adolphs and Tranel (1999)) or very high (as for anger in Adolphs and Tranel (1999)), we used a validated stimulus set comprising low and high intensity of emotional expression.

## 2 Methods

### 2.1 Design

Task 1 was a multinomial emotion identification task, for comparison with the previous literature, previously validated on a large clinical sample (Bach, Buxtorf, Grandjean, & Strik, 2009). A subset of the stimuli (angry, fearful, and neutral) was used for the 2-alternative forced choice (2AFC) task 2. Task 1 followed a nested 6 (emotional category: anger, fear, disgust, surprise, happiness, neutral)×2 (emotion intensity: low, high)×2 (group) factorial design. Due to the construction of the initial stimulus set, stimuli for disgust and neutral were not intensity-graded. Task 2 followed a completely crossed 2 (emotions pair: neutral-fearful, neutral-angry)×2 (emotion intensity: low, high)×2 (group) factorial design.

### 2.2 Participants

AM (previously also labelled patient 1) and BG (patient 2) (Becker et al., 2012) are monozygous female twins diagnosed at the age of 12 with congenital Urbach–Wiethe disease (lipoid proteinosis) due to a *de novo* mutation (Becker et al., 2012). This disorder in some cases leads to specific calcification of the amygdala that is thought to encroach on this structure gradually over the course of childhood and adolescence (Newton, Rosenberg, Lampert, & O’Brien, 1971). Despite these lesions, both twins exhibit only minor deficits in a standard neuropsychological test battery (Talmi, Hurlemann, Patin, & Dolan, 2010). At the time this research was conducted, they were 35 years old. The calcified volumes on high-resolution computer assisted tomography images include the whole basolateral amygdala and most other amygdala nuclei, only sparing anterior amygdaloid and ventral cortical amygdaloid parts at an anterior level, as well as lateral and medial parts of the central amygdaloid nucleus and the amygdalo-hippocampal area at posterior levels.

For experiment 1, we compared the patients against a control group acquired in the context of a different study (Bach et al., 2009); comprising 25 healthy participants (13 male, 12 female) with an age (mean±standard deviation) of 35.4±13.1 years. For experiment 2, we collected a sample more closely matched to the patients; these were 16 healthy females with an age of 33.6±3.4 years.

### 2.3 Stimuli

*Task 1*: Stimuli were taken from a validated set of Banse & Scherer (1996). The original work was concerned with acoustic profiles in vocal emotion expression that addressed the emotions fear, sadness, anger, disgust, neutral affect, and happiness. In the original set, 12 professional actors vocalised the emotions. There were two sentences for each emotion and intensity level, and each sentence was vocalised twice in two different eliciting scenarios. From the whole set, items were selected on the basis of expert ratings by an independent group of 12 actors. Those items were then included in a recognition study with naive participants. In the recognition study, stimuli were also included from actors who did not perform well on all emotions. To minimise variance caused by low-level acoustic features, we used only stimuli from the two actors (one male, one female) who performed the whole set of emotions. Therefore, the stimulus set used in the present study comprised only a part of the original set. Nine additional stimuli vocalised by a different actor were used as practice items for experiment 1. Hence, there were eight items for each intensity level of intensity-graded emotions, for two actors, two sentences, and two scenarios. For neutral and disgust, there were two different items from each actor/sentence/scenario combination, adding up to 16 items, to keep the total number of items per emotion category constant. The sentences were ‘Hat sundig pron you venzy’ and ‘Fee gott laish jonkill gosterr ’. These meaningless sentences comprise phonemes from several Indo-European languages and resemble normal speech. According to the validation study, ‘listeners generally have the impression of listening to an unknown foreign language’ (Banse & Scherer, 1996). Thus, experiment 1 used 96 stimuli expressing fear, sadness, anger, disgust, neutral affect, and happiness. Only stimuli for fear, sadness, anger, and happiness were graded in two intensity categories. Hence, a first analysis was performed on all six emotion categories while not accounting for intensity, and a second analysis on the four intensity-graded emotion categories.

*Task 2*: Stimuli for the second task were the subset of 16 angry, 16 fearful, and 16 neutral items from task 1.

### 2.4 Apparatus and procedure

*Task 1:* All stimuli were played on a standard PC, using eprime software (Psychology Software Tools, Pittsburgh PA, USA). Listeners could adjust the loudness ad libitum. Each stimulus was about 2 s in length. Stimuli were presented in randomized order. Participants responded by selecting the appropriate emotion category with a computer mouse. They had as much time to respond as they needed, but the presentation could not be repeated.

*Task 2:* Stimuli were played on a standard PC, using Matlab software (MathWorks, Natick MA, USA), with the Cogent toolbox (www.vislab.ucl.ac.uk). Each stimulus was presented once in each of two response contexts for 2 s in randomized order. Afterwards, participants were required to choose from a pair of emotions (fearful-neutral, angry-neutral, fearful-angry). Angry/fearful pairs were included in order to not bias the selection of the neutral response as a default response, without specific hypotheses. These were not included in the main analysis. Exploratory inclusion into the analysis of sensitivity did not result in any additional effects involving group, and there were no significant effects involving group in an intensity×group ANOVA of sensitivity only involving these pairs.

### 2.5 General procedure

Because patients performed both tasks one after the other, whereas control participants received only one of the tasks, we balanced task order in the patients to control for training effects. BG received first task 1, then task 2; AM received first task 2, then task 1.

### 2.6 Statistical analysis

Data extraction was implemented using R and Matlab. In task 1, we computed a measure of accuracy as hit rate for each emotion category. For task 2, we computed a measure of sensitivity as *d′=Z(hit rate)−Z(false alarm rate)*, and a measure of the response criterion, as *c=.5×(Z(hit rate)+Z(false alarm rate))* where *Z* is the quantile function of the standard normal distribution. Preliminary statistical analysis to localise effects was implemented in SPSS 20, using repeated-measures ANOVA in the General Linear Model routine, assuming equal variance. For interaction effects involving group, this approach might inflate type I error if variance in the control population is unequal between cells (Crawford, Garthwaite, & Howell, 2009); hence significant results were confirmed on a single case level in a Bayesian approach using Crawford's single case tests (Crawford & Garthwaite, 2007) as implemented in the authors' program dissocsbayes.exe. Non-significant results in the ANOVA approach do not require confirmation.

## 3 Results

**Fig. 1:**
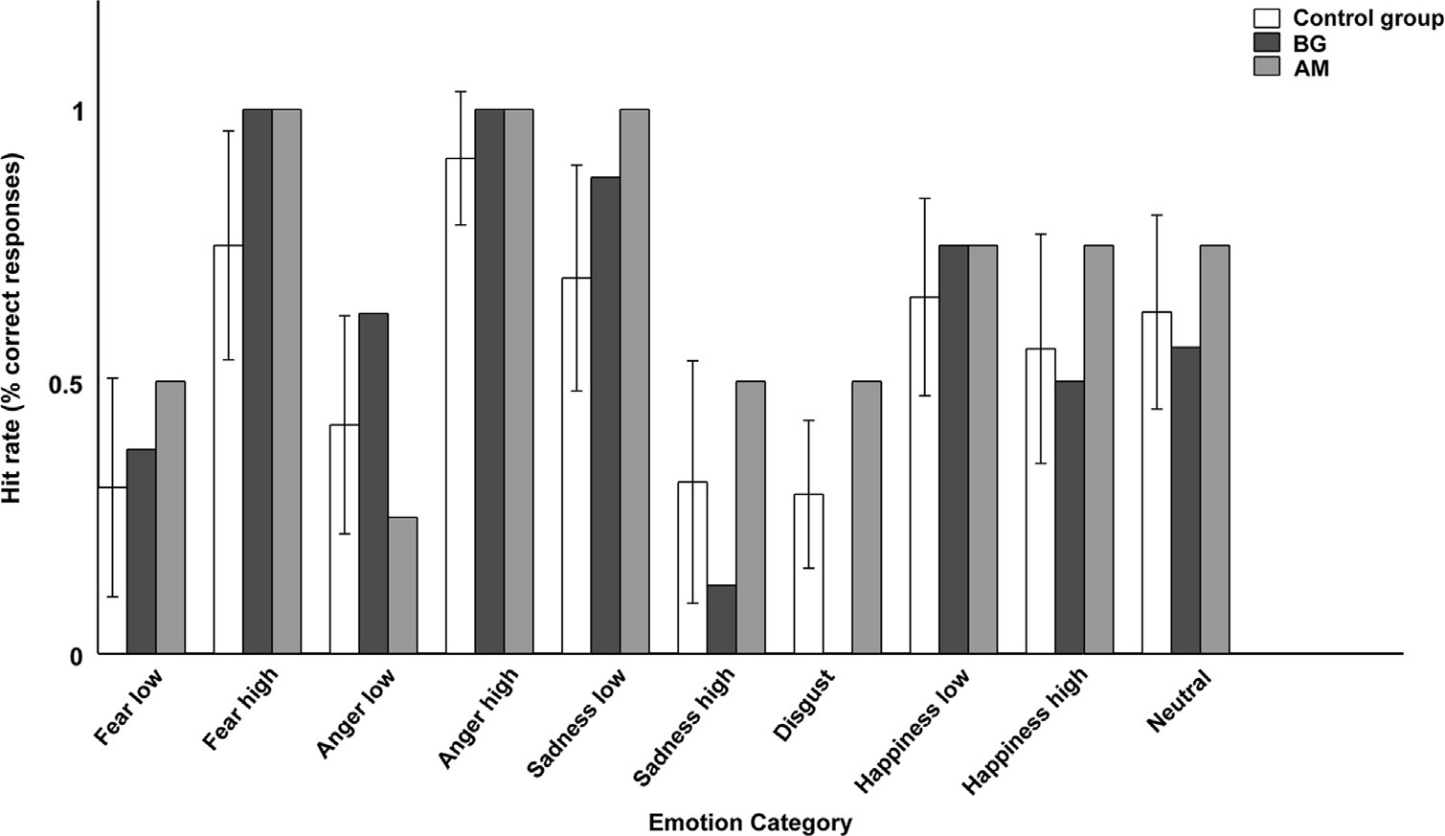
Recognition accuracy for the different emotion categories in a multinomial classification task (experiment 1). The graph shows mean±standard deviation for the control group, and individual results for patients BG and AM.

*Task 1*: This was a multinomial classification task; accuracy results (i. e. hit rates) for control group and patients are summarised in Fig. 1. Intensity-graded emotions were averaged, and data were analysed in a 6 (emotion)×2 (group) ANOVA. A main effect of emotion emerged (*η*^2^=.239, *F*(5, 125)=7.8, *p*<.001), but no effect involving group. Next, we analysed only the intensity-graded emotions in a 4 (emotion)×2 (intensity)×2 (group) ANOVA. We observed main effects of emotion (*η*^2^=.114, *F*(3, 75)=3.2, *p*<.05), intensity (*η*^2^=.558, *F*(1, 25)=35.6, *p*<.001), and emotion×intensity (*η*^2^=.569, *F*(3, 75)=33.00, *p*<.001), but no effects involving group.

Fig. 1 shows that there was no overall impairment for the patients; indeed it even appears that descriptively, they performed slightly better than the average of the control group, on high fear and low sadness. Note that in the control group, high sadness was recognized less often than low sadness; this had already been observed in the validation study (Banse & Scherer, 1996).

*Task 2*: Compared to the matched control sample, patients could more accurately (*d*′) discriminate between low-intensity fearful and neutral stimuli (Tables 1 and 2), as indicated by a trend-level (*p*=.06) significant interaction emotion×intensity×group, while they were only marginally different from controls for low-intensity angry and neutral stimuli, and for high-intensity stimuli of both emotion pairs. This group effect was clearly driven by patient AM.

**Fig. 2:**
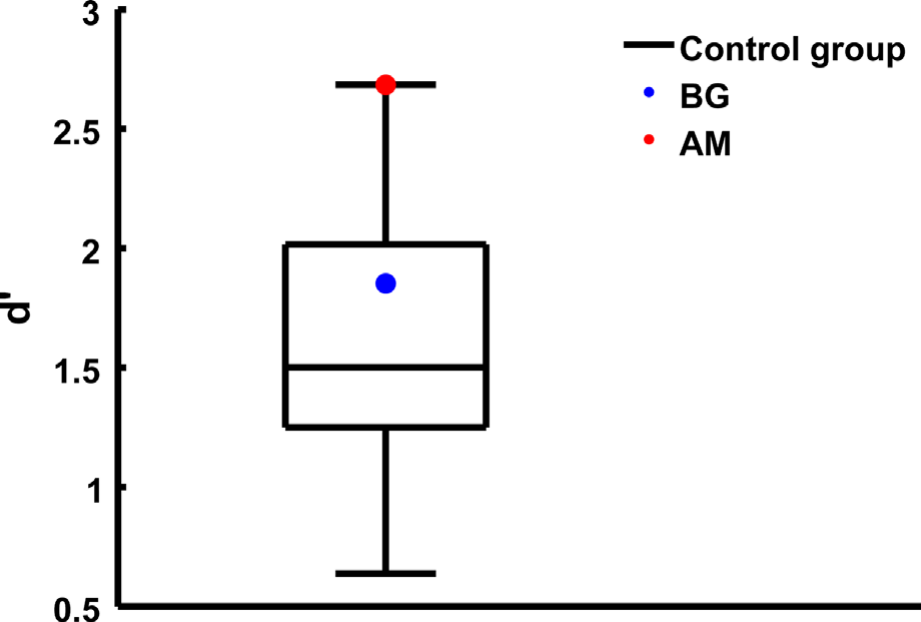
Discrimination (*d*′) between neutral and low-intensity fearful prosody. The graph shows a boxplot for the control individuals, and the patients as coloured dots. BG is better than the median of the control group, and AM performs as well as the control individual with the best performance in this task.

Comparing the patients separately to the control population demonstrated a highly significant (*p*<.01) effect for AM, and no effect for BG (Fig. 2). Accuracy (mean hit rate for both emotions presented with the same response context) shows that in the high-intensity condition, all participants were performing nearly at ceiling with much lower variance than in the low-intensity conditions, possibly explaining the lack of a group difference here. Because this unequal variance might inflate type I error in the ANOVA model for interaction effects involving group, we re-analysed the data after exclusion of the high intensity conditions. This confirmed a significant interaction emotion×group for AM (*η*^2^=.351, *F*(1, 15)=8.12, *p*=.012). Further, a Bayesian single case approach confirmed a significant dissociation between the *d'*-values for low anger and low fear for AM, as compared with the control group (*p*=.009), and a significantly higher value of *d′* for low fear than in the control group (*p*=.041).

No other effect involving group was significant in any model, and there was no effect in the analysis of the response criterion. Note that task order was balanced for the two patients, such as to rule out order effects. Patient AM did task 2 first, then task 1. Hence, this patient was in the same position as the control group with respect to training on the stimulus set and is thus better comparable to the control group on this task than patient BG. Training effects can therefore be firmly ruled out to underly the better discrimination ability in this patient.

## 4 Discussion

Whether impaired recognition of fearful face expression after selective amygdala lesions is due to a general deficit in recognition of fear in others, or due to a deficit specific to face perception, is currently unknown. Prosody is an important channel for conveying an emotional state but to date lesion studies using multinomial classification tasks have provided conflicting data on amygdala involvement in prosody recognition. Here, we assessed the ability of two patients with selective amygdala lesions to discriminate fearful/neutral, and angry/neutral prosody in a 2AFC task, thus separating response criterion and sensitivity (*d*′).

We find that one patient, AM, is significantly better in discriminating fearful and neutral prosody than a control group, matched for age and gender, while another, BG, is unimpaired. At the same time, both patients were not impaired in discriminating angry/neutral prosody. In a multinomial classification task which is less sensitive but allows comparison with previous literature, both patients did not differ from the control sample.

This is the second report of prosody recognition in patients with selective congenital amygdala lesions. Our results are in line with a previous report (Adolphs & Tranel, 1999), and with the view that impairments in recognising fearful prosody previously reported in some (Adolphs & Tranel, 1999; Brierley et al., 2004; Dellacherie et al., 2011; Scott et al., 1997; Sprengelmeyer et al., 1999) – but not all (Adolphs et al., 2001; Anderson & Phelps, 1998) – patients with unselective temporal lobe lesions are likely to be caused by damage to extra-amygdalar structures. A case of rather selective surgical amygdala lesions with prosody recognition impairment might also be explained by accidental damage to surrounding tissue, although insufficient data exists as to which precise structure might be responsible for the observed deficit (Scott et al., 1997). Although the patients in our study were not impaired in a multinomial classification task comprising several emotion categories, we cannot rule out that impairments might exist for emotions other than anger or fear, for which we did not assess emotion discrimination in a sensitive approach. If such deficits exist, it would however be difficult to relate them to the recognition deficit for fearful face expression seen in patients with amygdala lesions, in any case arguing against a domain-unspecific fear recognition deficit.

We note that in both patients, some minor parts of the amygdala were not calcified and it is possible that intact neuronal tissue exists in this area. We cannot fully rule out a possibility that this possibly remaining functionally active tissue supports fearful prosody recognition. Also, other brain structures might compensate for possible deficits, and even over-compensate and thus support the better-than-normal performance for AM.

While functional magnetic resonance imaging studies have consistently reported amygdala activation following non-verbal emotional vocalisation (Fecteau, Belin, Joanette, & Armony, 2007; Morris, Scott, & Dolan, 1999; Phillips et al. 1998; Sander & Scheich, 2001; Seifritz et al. 2003) or simple auditory warning cues (Bach et al., 2008), emotional prosody in verbalisation appears to generate less robust amygdala responses, and in particular not to fearful prosody (see (Wiethoff et al., 2009) for a rare report of amygdala responses to fearful prosody which were however the least pronounced responses of all assessed emotion categories). This is in keeping with the present finding. However, many have found stronger amygdala responses to angry as opposed to neutral prosody (Ethofer et al., 2009; Fruhholz et al., 2012; Fruhholz & Grandjean, 2012; Grandjean et al., 2005; Mothes-Lasch et al., 2011; Wiethoff et al., 2009). Amygdala BOLD responses to angry prosody might therefore be regarded a robust finding, but we note that the amygdala does not appear to be critically involved in the discrimination or recognition of angry prosody either, as revealed by both experiments reported here.

If the amygdala has no function in recognising fearful prosody, why would a patient with amygdala lesion show enhanced rather than normal fearful/neutral prosody discrimination? There are several answers to this question. First, we note that there is variance in the control sample, and AM performed similar to the best control participant. This could imply that some, perhaps non-genetic, individual characteristics entirely unrelated to an amygdala lesion determines prosody discrimination, a possibility that would explain the discrepany between the two twin sisters studied here. On the other hand, given an impairment in recognising fearful face expression commonly observed in patients with amygdala lesions, it is conceivable that capitalising on fearful prosody provides a simple compensatory strategy (Mihov et al., 2013) such that prolonged experience-dependent training improves performance in such a task. We note however that AM is currently not grossly impaired in recognising fearful face expression (Becker et al., 2012), though her ability to recognise fearful body posture (Dael et al., 2012) has not been assessed as yet. In essence, any interpretation of AM's better performance remains speculative at present.

The fact that we – and others (Adolphs & Tranel, 1999) – found no evidence for amygdala involvement in recognising fearful prosody argues against a domain-unspecific function of the amygdala for extraction of a conspecific's fearful state. This is complemented by a report on patients SM and AP that the amygdala is not crucial for recognition of fearful body posture either (Atkinson, Heberlein, & Adolphs, 2007). To summarise, impaired recognition of fearful face expression after amygdala lesions might be a rather specific deficit, confined to face expression, without generalisation to other sources of emotional information. As compensating mechanisms might explain the current results even if the amygdala had a role in discriminating fearful prosody, findings from studies using other modalities would underline the current observations.

## Figures and Tables

**Table 1:** Task 2: *d*′, response criterion, and accuracy (combined hit rate) for each of the three emotion combinations. Criterion is coded using hit rate for the first of the two emotions, and false alarm rate for the second.

			**Fear – Neutral**		**Anger – Neutral**		**Fear – Anger**	
			Low	High	Low	High	Low	High
***d***′	**Control**	M	1.62	3.02	2.05	2.90	2.21	2.94
		SD	*.55*	*.13*	*.59*	*.35*	*.40*	*.29*
	**BG**		1.85	2.68	2.68	3.07	1.85	3.07
	**AM**		2.68	3.07	1.47	3.07	2.21	3.07
**Criterion**	**Control**	M	−.25	−.02	−.10	.06	−.08	.04
		SD	*.36*	*.07*	*.34*	*.16*	*.32*	*.11*
	**BG**		−.61	−.19	−.19	.00	.61	.00
	**AM**		.19	.00	−.42	.00	−.43	.00
**Accuracy in %**	**Control**	M	77.34	99.22	84.38	97.27	86.72	98.05
		SD	*9.09*	*2.13*	*9.13*	*5.58*	*5.98*	*4.40*
	**BG**		81.25	93.75	93.75	100.00	81.25	100.00
	**AM**		93.75	100.00	75.00	100.00	87.50	100.00

**Table 2:** Task 2: ANOVA tables for *d*′ and response criterion as dependent measures, using a Emotion (E: Fear – Neutral, Anger – Neutral)×Intensity (I: Low, High)×Group (G: Patient, Control) model. We present results from pooled patients, followed by models for individual patients.

Effect	*d*′						Response criterion					
	Patients pooled		BG		AM		Patients pooled		BG		AM	
	*η*^2^	*F* (1, 16)	*η*^2^	*F* (1, 15)	*η*^2^	*F* (1, 15)	*η*^2^	*F* (1, 16)	*η*^2^	*F* (1, 15)	*η*^2^	*F* (1, 15)
*E*	.013	<1	.190	3.5	.079	1.3	.020	<1	.143	2.5	.032	<1
*I*	.617	25.8⁎⁎⁎	.413	10.6⁎⁎	.512	15.8⁎⁎⁎	.170	3.3	.044	2.5	.060	<1
*E*×*I*	.008	<1	.177	3.2	.088	1.5	.013	<1	.38	<1	.127	2.2
*G*	.047	<1	.026	<1	.26	<1	.029	<1	.082	1.3	.001	<1
*G*×*E*	.013	<1	.079	1.3	.190	3.5	.020	<1	.032	<1	.142	2.5
*G*×*I*	.044	<1	.059	<1	.004	<1	.000	<1	.008	<1	.005	<1
*G*×*E*×*I*	.213	4.3(⁎^)^	.002	<1	.403	10.1⁎⁎	.048	<1	.013	<1	.180	3.3

(⁎) *p*=.06.

⁎⁎ *p*<.01.

⁎⁎⁎ *p*<.001.

## References

[bib1] Adolphs R., Damasio H., Tranel D. (2002). Neural systems for recognition of emotional prosody: A 3-D lesion study. Emotion.

[bib2] Adolphs R., Tranel D. (1999). Intact recognition of emotional prosody following amygdala damage. Neuropsychologia.

[bib3] Adolphs R., Tranel D., Damasio H. (2001). Emotion recognition from faces and prosody following temporal lobectomy. Neuropsychology.

[bib4] Adolphs R., Tranel D., Hamann S., Young A.W., Calder A.J., Phelps E.A. (1999). Recognition of facial emotion in nine individuals with bilateral amygdala damage. Neuropsychologia.

[bib5] Anderson A.K., Phelps E.A. (1998). Intact recognition of vocal expressions of fear following bilateral lesions of the human amygdala. Neuroreport.

[bib6] Atkinson A.P., Adolphs R. (2011). The neuropsychology of face perception: beyond simple dissociations and functional selectivity. Philosophical Transactions of the Royal Society of London B, Biological Sciences.

[bib7] Atkinson A.P., Heberlein A.S., Adolphs R. (2007). Spared ability to recognise fear from static and moving whole-body cues following bilateral amygdala damage. Neuropsychologia.

[bib8] Bach D.R., Buxtorf K., Grandjean D., Strik W.K. (2009). The influence of emotion clarity on emotional prosody identification in paranoid schizophrenia. Psychological Medicine.

[bib9] Bach D.R., Grandjean D., Sander D., Herdener M., Strik W.K., Seifritz E. (2008). The effect of appraisal level on processing of emotional prosody in meaningless speech. Neuroimage.

[bib10] Bach D.R., Schachinger H., Neuhoff J.G., Esposito F., Salle F.D., Lehmann C. (2008). Rising sound intensity: an intrinsic warning cue activating the amygdala. Cerebral Cortex.

[bib11] Banse R., Scherer K.R. (1996). Acoustic profiles in vocal emotion expression. Journal of Personality and Social Psychology.

[bib12] Becker B., Mihov Y., Scheele D., Kendrick K.M., Feinstein J.S., Matusch A. (2012). Fear processing and social networking in the absence of a functional amygdala. Biological Psychiatry.

[bib13] Breiter H.C., Etcoff N.L., Whalen P.J., Kennedy W.A., Rauch S.L., Buckner R.L. (1996). Response and habituation of the human amygdala during visual processing of facial expression. Neuron.

[bib14] Brierley B., Medford N., Shaw P., David A.S. (2004). Emotional memory and perception in temporal lobectomy patients with amygdala damage. Journal of Neurology, Neurosurgery, and Psychiatry.

[bib15] Crawford J.R., Garthwaite P.H. (2007). Comparison of a single case to a control or normative sample in neuropsychology: Development of a Bayesian approach. Cognitive Neuropsychology.

[bib16] Crawford J.R., Garthwaite P.H., Howell D.C. (2009). On comparing a single case with a control sample: An alternative perspective. Neuropsychologia.

[bib17] Dael N., Mortillaro M., Scherer K.R. (2012). Emotion expression in body action and posture. Emotion.

[bib18] Dellacherie D., Hasboun D., Baulac M., Belin P., Samson S. (2011). Impaired recognition of fear in voices and reduced anxiety after unilateral temporal lobe resection. Neuropsychologia.

[bib19] Dolan R.J., Morris J.S., de Gelder B. (2001). Crossmodal binding of fear in voice and face. Proceedings of the National Academy of Sciences of the USA.

[bib20] Ekman P., Oster H. (1979). Facial expressions of emotion. Annual Review of Psychology.

[bib21] Ethofer T., Kreifelts B., Wiethoff S., Wolf J., Grodd W., Vuilleumier P. (2009). Differential influences of emotion, task, and novelty on brain regions underlying the processing of speech melody. Journal of Cognitive Neuroscience.

[bib22] Fecteau S., Belin P., Joanette Y., Armony J.L. (2007). Amygdala responses to nonlinguistic emotional vocalizations. Neuroimage.

[bib23] Fischer H., Wright C.I., Whalen P.J., McInerney S.C., Shin L.M., Rauch S.L. (2003). Brain habituation during repeated exposure to fearful and neutral faces: a functional MRI study. Brain Research Bulletin.

[bib24] Fruhholz S., Ceravolo L., Grandjean D. (2012). Specific brain networks during explicit and implicit decoding of emotional prosody. Cerebral Cortex.

[bib25] Fruhholz S., Grandjean D. (2013). Amygdala subregions differentially respond and rapidly adapt to threatening voices. Cortex.

[bib26] Grandjean D., Sander D., Pourtois G., Schwartz S., Seghier M.L., Scherer K.R. (2005). The voices of wrath: Brain responses to angry prosody in meaningless speech. Nature Neuroscience.

[bib27] Mihov Y., Kendrick K.M., Becker B., Zschernack J., Reich H., Maier W. (2013). Mirroring fear in the absence of a functional amygdala. Biological Psychiatry.

[bib28] Morris J.S., Frith C.D., Perrett D.I., Rowland D., Young A.W., Calder A.J. (1996). A differential neural response in the human amygdala to fearful and happy facial expressions. Nature.

[bib29] Morris J.S., Scott S.K., Dolan R.J. (1999). Saying it with feeling: neural responses to emotional vocalizations. Neuropsychologia.

[bib30] Mothes-Lasch M., Mentzel H.J., Miltner W.H., Straube T. (2011). Visual attention modulates brain activation to angry voices. Journal of Neuroscience.

[bib31] Newton F.H., Rosenberg R.N., Lampert P.W., O'Brien J.S. (1971). Neurologic involvement in Urbach–Wiethe's disease (lipoid proteinosis). A clinical, ultrastructural, and chemical study. Neurology.

[bib32] Phillips M.L., Young A.W., Scott S.K., Calder A.J., Andrew C., Giampietro V. (1998). Neural responses to facial and vocal expressions of fear and disgust. Proceedings of the Royal Society B: Biological Sciences.

[bib33] Sander K., Scheich H. (2001). Auditory perception of laughing and crying activates human amygdala regardless of attentional state. Brain Research—Cognitive Brain Research.

[bib34] Scott S.K., Young A.W., Calder A.J., Hellawell D.J., Aggleton J.P., Johnson M. (1997). Impaired auditory recognition of fear and anger following bilateral amygdala lesions. Nature.

[bib35] Seifritz E., Esposito F., Neuhoff J.G., Luthi A., Mustovic H., Dammann G. (2003). Differential sex-independent amygdala response to infant crying and laughing in parents versus nonparents. Biological Psychiatry.

[bib36] Sprengelmeyer R., Young A.W., Schroeder U., Grossenbacher P.G., Federlein J., Buttner T. (1999). Knowing no fear. Proceedings: Biological Sciences.

[bib37] Talmi D., Hurlemann R., Patin A., Dolan R.J. (2010). Framing effect following bilateral amygdala lesion. Neuropsychologia.

[bib38] Wagner H.L. (1993). On measuring performance in category judgment studies of nonverbal behavior. Journal of Nonverbal Behavior.

[bib39] Whalen P.J., Kagan J., Cook R.G., Davis F.C., Kim H., Polis S. (2004). Human amygdala responsivity to masked fearful eye whites. Science.

[bib40] Whalen P.J., Rauch S.L., Etcoff N.L., McInerney S.C., Lee M.B., Jenike M.A. (1998). Masked presentation of emotional facial expressions modulate amygdala activation without explicit knowledge. Journal of Neuroscience.

[bib41] Wiethoff S., Wildgruber D., Grodd W., Ethofer T. (2009). Response and habituation of the amygdala during processing of emotional prosody. Neuroreport.

